# Pharmacoeconomics and its implication on priority-setting for essential medicines in Tanzania: a systematic review

**DOI:** 10.1186/1472-6947-12-110

**Published:** 2012-09-27

**Authors:** Amani Thomas Mori, Bjarne Robberstad

**Affiliations:** 1 Centre for International Health, University of Bergen, P.O. Box 7804, 5020 Bergen, Norway; 2 Muhimbili University of Health and Allied Sciences, School of Pharmacy, P.O. Box 65013, Dar es Salaam, Tanzania

**Keywords:** Tanzania, Essential medicines, Pharmacoeconomics, Cost-effectiveness, Priority-setting, National essential medicine list, Decision-making, Disease burden, Low-income countries

## Abstract

**Background:**

Due to escalating treatment costs, pharmacoeconomic analysis has been assigned a key role in the quest for increased efficiency in resource allocation for drug therapies in high-income countries. The extent to which pharmacoeconomic analysis is employed in the same role in low-income countries is less well established. This systematic review identifies and briefly describes pharmacoeconomic studies which have been conducted in Tanzania and further assesses their influence in the selection of essential medicines.

**Methods:**

Pubmed, Embase, Cinahl and Cochrane databases were searched using “economic evaluation”, “cost-effectiveness analysis”, “cost-benefit analysis” AND “Tanzania” as search terms. We also scanned reference lists and searched in Google to identify other relevant articles. Only articles reporting full economic evaluations about drug therapies and vaccines conducted in Tanzania were included. The national essential medicine list and other relevant policy documents related to the identified articles were screened for information regarding the use of economic evaluation as a criterion for medicine selection.

**Results:**

Twelve pharmacoeconomic studies which met our inclusion criteria were identified. Seven studies were on HIV/AIDS, malaria and diarrhoea, the three highest ranked diseases on the disease burden in Tanzania. Six studies were on preventive and treatment interventions targeting pregnant women and children under the age of five years. The national essential medicine list and the other identified policy documents do not state the use of economic evaluation as one of the criteria which has influenced the listing of the drugs.

**Conclusion:**

Country specific pharmacoeconomic analyses are too scarce and inconsistently used to have had a significant influence on the selection of essential medicines in Tanzania. More studies are required to fill the existing gap and to explore whether decision-makers have the ability to interpret and utilise pharmacoeconomic evidence. Relevant health authorities in Tanzania should also consider how to apply pharmacoeconomic analyses more consistently in the future priority-setting decisions for selection of essential medicines.

## Background

Pharmacoeconomic analysis is the comparison of costs and consequences of alternative drug therapies so as to maximize therapeutic outcomes when resources are limited. Use of pharmacoeconomics is important in priority-setting between drug therapies since budgets are finite and there is great variance in value for money for products in the market. Some products are more costly but add little or no extra benefits when compared to the existing drug therapies. In other situations new and more expensive drugs represent large potential health improvements. Pharmacoeconomic evidence can help decision-makers judge whether the therapeutic benefits produced by a new drug are worth the extra costs [1].

In high-income countries pharmacoeconomic analysis is widely used to guide priority-setting decisions for pharmaceuticals [2]. National Institute of Clinical Excellence (NICE) in the UK and the Canadian Agency for Drugs and Technology in Health (CADTH) are examples of institutions which have been established for pharmacoeconomic evaluation of new pharmaceutical products and technologies [3,4]. Pharmacoeconomic evaluation has also gained acceptance at hospital level in formulary decision-making in these countries [5]. By contrast, in low-income countries applied economic evaluation studies are not only scarce, but their usefulness on essential medicine selection has also been debated in the literature [6,7].

Essential medicines are those which address priority healthcare needs of the populations. Since its inception, the concept of essential medicines aims to increase availability and accessibility of medicines in low-income countries [8]. The strategy was consolidated in the Alma Ata conference where access to essential medicines was listed as one of the key component of the primary healthcare package [9]. Increase in access to high quality essential medicines is today viewed as the most important global strategy to reduce the burden of diseases [10]. This strategy is of particular importance for low-income countries which carry a disproportionately large share of the disease burden [11], but yet are accounted for as little as one per cent of the total global pharmaceutical expenditures [12].

Tanzania had its first national essential medicine list in 1991, while the current edition of 2007 is the third in the series. The national essential medicine list is considered to be in line with the WHO recommendations under the Tanzania conditions [13]. WHO proposed the use of evidence-based approach in the selection process of essential medicines, with cost-effectiveness comparisons being one of the key criteria [14]. Little country specific cost-effectiveness evidence is available for Tanzania [15], which raises questions on whether, how and to what extent such evidence is actually used to guide priority-setting decisions. Therefore this systematic review aims to identify and briefly describe pharmacoeconomic studies which have been conducted in Tanzania and assess their influence on the priority-setting process for selection of essential medicines.

## Methods

We used the PRISMA checklist which is suited for reporting systematic review of randomized trials but also recommended for other systematic review studies [16]. Some modifications were done to adopt the checklist to report economic evaluation studies.

### Information sources

Pubmed and Cinahl databases were searched for all years, limiting the search to English language using the combinations of the following search terms: “economic evaluations”, “cost-effectiveness analysis”, “cost-benefit analysis” AND “Tanzania”. Cochrane library was searched using the key word “Tanzania” in its NHS economic evaluation databases, and using “cost-effectiveness analysis” AND “Tanzania” in its Cochrane Control Register of Controlled Trials Database. Embase was searched from 1980 to 2011(week 51) limiting the search to English language and “Human”. “Economic evaluations” AND “Tanzania”, “cost-effectiveness analysis” AND “Tanzania” and “cost-benefit analysis” AND “Tanzania” were used as search terms. Last search of these databases was 30^th^ December 2011. Other articles were identified by scanning reference lists and searching by Google search engine using the above mentioned search terms.

The Tanzanian national essential medicine list and other relevant policy documents related to the identified articles were also screened for information related to the use of economic evaluation evidences as a criterion for the selection of the recommended medicines. Also we aimed to determine whether the medicines listed in these policy documents were similar to those recommended by the authors of the articles we had identified.

### Study selection criteria and rationales

#### Inclusion criteria

1. Study design: economic evaluation since the aim was to compare costs and outcomes of alternative interventions competing for the same resources

2. Study interventions: drug therapies or vaccines only since these are the ones listed on treatment guidelines and national essential medicine list

3. Study setting: Tanzania

4. Publication type: Original full articles or reports

#### Exclusion criteria

1. Economic evaluation studies of the methods used to distribute the drugs or vaccines to the patients since this was not our study focus

2. Studies presenting only costs or only effectiveness results since they provide insufficient information required for cost-effectiveness assessment

3. Hypothetical interventions since they do not represent actual intervention strategies

4. Review articles since they contain information extracted from individual studies already included

Each article was initially screened based on its title and the abstract to see whether it met our inclusion and exclusion criteria. Articles which passed the screening stage were subjected to full text assessment for eligibility. Eligible articles were selected for the qualitative analysis.

### Data extraction procedure

Necessary information such as names of the authors, publication year, the target intervention, study perspectives and the recommended drug therapies and their cost-effectiveness ratios were extracted from each of the twelve articles. Ranking of the disease burden was extracted from the Tanzania national package of the essential health interventions. Generic names of the recommended drugs and vaccines and the rationales behind them, were extracted from the national essential medicine list and other relevant policy documents.

## Results

### Study selection

**Figure 1:**
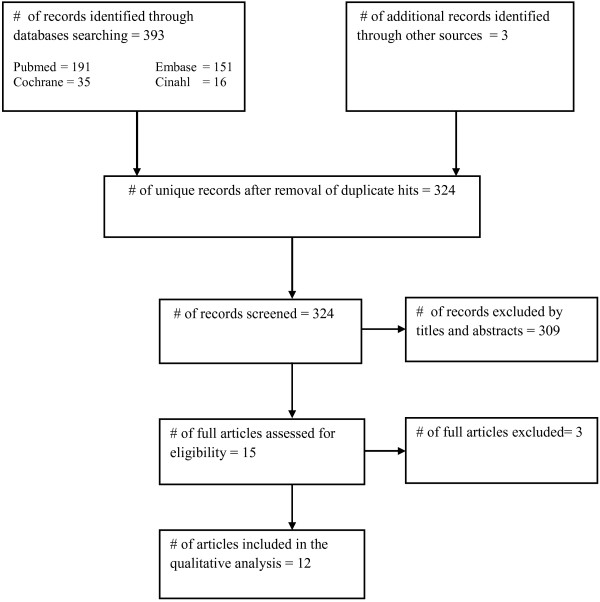
**Flow of information through the different phases of the systematic review.**

396 articles were retrieved from various databases and other sources in which 72 were excluded because they were duplicate hits. The remaining 324 unique articles were screened by titles and abstracts after which 309 articles were excluded. Three articles out of the remaining 15 were excluded because one was a brief communication [17], the second was about a hypothetical malaria vaccine [18], and the third was a review study [19]. Therefore only 12 full articles qualified for the qualitative analysis [20-31] (Figure 1) .

### Burden of diseases versus availability of pharmacoeconomic studies

Tanzania has a list of twelve priority disease conditions referred to as a national package of essential health interventions, on which to prioritize the allocation of its scarce resources for health. This list rank disease conditions according to their burden of disease and is dominated by infectious diseases – HIV/AIDS, malaria and diarrhoeal diseases are at the top. Ranking of the disease conditions is fairly consistent with the number of pharmacoeconomic studies we have identified. Nine out of the twelve pharmacoeconomic analysis studies addresses the four highest ranked disease conditions (Table 1) It is disappointing to note that only one pharmacoeconomic study addresses non-communicable diseases, and none are available for acute respiratory tract infections, diabetes, cancers, and nutritional deficiencies.

**Table 1:** **Disease burden rank, pharmacoeconomic evidences and their main findings, implications and current listing status**

**Rank**	**Disease**	**Tanzanian pharmacoeconomic evidence**	**Main findings, implications and current listing status**
1	HIV/AIDS	HAART for PMTCT [21]	Highly cost-effective intervention with ICER of US$ 162 per DALY averted when compared to sd-NVP, however it is 40% more costly but 5 times more effective
			*(* Listing status: HAART *is one of the two options recommended by WHO but not the one being implemented in Tanzania, an area for future research)*
		Sd-NVP for PMTCT [24]	*(*Listing status: Use of Sd-NVP *is the old policy which was also based on WHO’s recommendations but currently being phased out in Tanzania)*
2	Malaria	ALu for non-severe malaria [26]	A cost-effective drug which saves US$ 22.4 per case averted when compared to amodiaquine. (Listing status: ALu *is one of the few artemisinin-based combination therapies recommended by WHO and is the current drug of choice in Tanzania*)
		SP for non-severe malaria [25]	*(*Listing status: *Use of SP was replaced by ALu since 2007 due to parasite resistance but still listed as essential medicine for IPTp)*
		SP for IPTi [27]	A cost-effective intervention with ICER of US$ 1.6-12.2 per DALY^*^ averted. SP-IPTi reduces episodes of clinical malaria and anaemia by 30 and 21 percent in areas of moderate to high malaria transmissions, in the first year of life [32].
			*(*Listing status: *SP-IPTi is a new intervention strategy recommended by WHO since 2010 but not yet adopted in Tanzania)*
3	Diarrhoeal diseases	Zinc as adjunct therapy [23]	A highly cost-effective intervention when combined with ORS with ICER of US$ 73 per DALY averted
			*(*Listing status: *Listed on essential medicine list since 2007, based on WHO’s recommendations)*
4	Injury/ Trauma	Tranexamic acid Inj for surgical bleeding and trauma patients [20,29]	A highly cost-effective intervention with ICER of US$ 93 and US$ 48 per life saved for surgical and trauma patients^*^. TXA reduces number of transfusions by one-third and volume of blood per transfusion by one unit in elective surgery [33]. TXA reduces risks of death by 21% if administered within 3 hrs after injury [34].
			*(*Listing status: Tranexamic acid Inj. *was listed recently on WHO’s list of essential medicine but not yet listed in Tanzania)*
5	ARI	None	None
6	TB	Short-course chemotherapy [31]	A highly cost-effective option with ICER of US$ 1–4 per LY saved. Short-course chemotherapy increases cure rate by 25% compared to the long regimens.
			*(*Listing status: *Listed; Introduced and adopted in Tanzania in mid 1980’s)*
7	Prenatal conditions	None	None
8	Maternal deficiencies	None	None
9	Nutritional deficiencies	Iron+ Deltaprim to prevent anaemia and malaria in infants [28]	Considered to be a cost-effective intervention, support the evidence shown by SP-IPTi in reduction of both anaemia and malaria
			*(*Listing status: Deltaprin (dapsone +pryrimethamine) *is not listed as essential medicine in Tanzania*
10	CVD and Diabetes	Preventive cardiology [22]	Diuretics, Aspirin+Diuretic and Aspirin+Diuretic+β-blocker are very cost-effective with ICERS of US$ 85, 143 and 317 per DALYS averted.
			*(*Listing status *: new evidence but these drugs were already listed as essential medicines before the publication of the study)*
11	Neoplasms	None	None
12	Immunisable diseases	Anti-Rabies vaccine [30]	A very cost-effective intervention with ICER of US$ of 27 and 32 per DALY^*^ averted from provider and societal perspectives.
			*(*Listing status: *New evidence, but the vaccine was already listed as essential medicine before the publication of the study)*

* Compared to do nothing, ALu-artemether-lumefantrine, SP- sulphadoxine-pyrimethamine, Sd-Single dose, HAART-Highly active antiretroviral drugs, ORS-Oral rehydration salt, ARI-acute respiratory tract infections, CVD-cardiovascular diseases.

## Discussion

The World Health Report has classified interventions with cost-effectiveness ratios of less than the country’s per capita GDP as highly cost-effective and those which are 1–3 times the per capita GDP as cost-effective [35]. Most of the interventions we have identified in this study have cost-effectiveness ratios which are well below the Tanzania’s estimated GDP per capita of US$ 550 [36], hence they can be considered as highly cost-effective. On the other hand, Tanzania has a per capita expenditure on health of about US$ 14 per year [37], which is below the US$ 40 recommended by WHO to finance essential health interventions [38]. This means its ability to implement and scale-up even what can be considered as a highly cost-effective intervention is limited.

Our literature review shows that only a few pharmacoeconomic studies have been conducted in Tanzania. Nine out of the twelve studies were on drug therapies and vaccine against infectious diseases which are responsible for more than two-third of the disease burden in sub-Saharan Africa [39]. Nine studies were published within the last ten years, of which six are less than five years old indicating an increasing focus on this research area (Table 2). Antimalarial and antiretroviral drugs were the most researched drugs, which mean that to some extent researchers have responded to the importance of the two diseases for the burden of diseases in Tanzania (Table 1). Half of the identified studies were on interventions targeting pregnant women and children under the age of five years, reflecting concerns for the high mortality rates for these vulnerable groups in Tanzania.

**Table 2:** Study characteristics

**Authors**	**Year**	**Target Interventions**
Guerriero et al. [20]	2011	Injury (Bleeding Trauma Patients)
Robberstad et al. [21]	2010	HIV/AIDS (Prevention of Mother-to-Child Transmissions)
Guerriero et al. [29]	2010	Surgical Bleeding
Hutton et al. [27]	2009	Malaria (Intermittent Prevention Therapy in Infants)
Shim et al. [30]	2009	Rabies vaccination
Robberstad et al. [22]	2007	Cardiovascular diseases
Wiseman et al. [26]	2006	Case management of non-severe malaria
Robberstad et al. [23]	2004	Diarrhoeal diseases
Sweat et al. [24]	2004	HIV/AIDS (Prevention of Mother-to-Child Transmissions)
Abdulla et al. [25]	2000	Case management of non-severe malaria
Gonzalez et al. [28]	2000	Malaria (Intermittent Prevention Therapy in Infants)
Murray et al. [31]	1991	Tuberculosis

### HIV/AIDS

HIV/AIDS is the number one priority health problem in Tanzania, and affects the most productive age group ranging from 15–59 years, hence impairing the country’s economic growth [40]. About 20 per cent of the mortalities for admitted patients above five years of age recorded in Tanzania each year are due to HIV/AIDS and Tuberculosis [41]. Our study found two pharmacoeconomic studies on prevention of mother-to-child transmission (PMTCT) and none on case management of HIV/AIDS.

PMTCT programs are in transition in Tanzania, responding to the current recommendations consisting of two prophylactic options provided by the WHO. Option A consists of zidovudine (AZT) which is initiated on week 14 of pregnancy, followed with single dose nevirapine (sd-NVP) plus lamivudine (3TC) at the onset of labour until delivery. AZT and 3TC are then continued for 7 days postpartum. Option B is composed of triple ARV drugs which are also initiated on week 14 of pregnancy until one week after cessation of breastfeeding [42]. The task of choosing which option to implement rests on individual countries and should be based on the feasibility, acceptability, safety and costs [42]. This is a practical example where pharmacoeconomic analysis should be used to guide medicine selection.

Tanzania has opted to implement option A [43], however, without being guided by cost-effectiveness comparison evidence for option A and B. An economic evaluation study by Robberstad et al. at Haydom Lutheran Hospital in Northern Tanzania showed that option B was highly cost-effective in the Tanzanian settings with incremental cost-effectiveness ratio of US$ 162 per DALY averted. This regimen was however 40 per cent more expensive than sd-NVP but 5 times more effective [21]. Since option A at the time of the study was not being implemented at the study site, they did not make cost-effectiveness comparisons of option A and B relative to sd-NVP. Drug costs for option B relative to option A which were approximately up to five times in 2009, have been reduced significantly down to two times by the end of 2011 [44]. WHO has recently released a new PMTCT update advising countries to adopt the use of option B plus, where a pregnant woman is placed on option B for life regardless of CD4 cell count or clinical staging [45].

### Malaria

Malaria is second after HIV/AIDS on the disease burden in Tanzania. On average about 46 per cent of all in-patient and out-patient cases registered in the healthcare facilities each year are due to malaria [41]. Malaria is the leading cause of morbidity and mortality among children under the age of five years [40,41]. Malaria during pregnancy is also associated with low birth weight [46], which is recognized as the single greatest risk factor for neonatal and infant mortalities in sub-Saharan countries [47]. A recent study showed that the burden of malaria among adults has been highly underestimated. According to the findings of this study, malaria is also the major cause of deaths among adult populations [48].

Our review found four pharmacoeconomic studies on malaria, two of them being on malaria case management. Tanzania has changed its national malaria treatment policy twice over the past ten years due to drug resistance to formerly effective antimalarials. These policy changes involved replacement of chloroquine (CQ) with sulphadoxine-pyrimethamine (SP), which was subsequently replaced by artemether-lumefantrine (ALu) [49,50]. Both SP and ALu were at the time the most cost-effective antimalarials compared to alternatives which were available [25,26]. Our review of treatment guidelines and other relevant policy documents showed inconsistent use of pharmacoeconomic evaluations during malaria treatment policy change. As a result the decision to change to ALu unlike that of changing to SP has been criticized for largely being based on the efficacy rather than cost-effectiveness comparisons [51].

The other two studies were on presumptive treatment of malaria using SP in infants (SP-IPTi) and Deltaprim (a combination of pyrimethamine and dapsone) plus Iron in infants and pregnant women. Studies from African settings have shown that SP-IPTi could reduce episodes of clinical malaria, anaemia and rates of hospitalization in infants by 30, 21 and 38 per cent respectively [32]. As a result SP-IPTi has been adopted by WHO since 2010 as a new malaria intervention strategy targeting infants residing in areas with moderate to high malaria transmissions, but with low resistance to SP [52]. SP-IPTi was demonstrated to be highly cost-effective in Tanzania with incremental cost-effectiveness ratios of US$ 1.57 (0.8-4.0) and US $ 3.7 (1.6-12.2) per malaria episode and DALY averted, respectively [27]. Even though Global Fund and other donors have made financial resources available to support the implementation of this intervention [53], SP-IPTi has not yet been adopted in Tanzania. Studies from the Northern and Southern areas of the country have reported low protective efficacy results from the use of this intervention [54,55].

### Diarrhoeal diseases

Diarrhoea is ranked third on the disease burden in Tanzania and is considered the second main cause of deaths among children under the age of five years worldwide after malaria [56]. Oral rehydration salts (ORS) reduce the duration of diarrhoea episode and replaces the lost water and electrolytes hence preventing the occurrence of dehydration. When Zinc is given as an adjunct therapy for 10–14 days, it has been proved to reduce the duration of acute diarrhoea by 25 per cent and treatment failure or death due to persistent diarrhoea by 42 per cent. It also prevents episodes of subsequent infections for up to three months [57,58]. In 2004, WHO and UNICEF recommended that countries adopt the use of Zinc and low osmolarity oral rehydration salts (lo-ORS) in their revised guidelines for treatment of diarrhoea [59]. Zinc was included in WHO model list of essential medicines in 2005 based on the evidence of cost, efficacy, safety and cost-effectiveness in the management of diarrhoea [60].

We found one pharmacoeconomic study by Robberstad et al. on Zinc as adjunct therapy which reported it to be cost-effective in Tanzania [23]. Tanzania adopted the new diarrhoea treatment guidelines which incorporated the use of Zinc in July 2007 [61] followed by its listing in the national essential medicine list the same year [13]. Our review of documents revealed that a task force committee which was composed of representatives from the government, WHO, UNICEF, and non-governmental organization was formed to advocate for adoption of Zinc [61]. However there is no evidence of whether economic evaluation was among the criteria on which the local decision was based apart from the WHO/UNICEF recommendation.

### Injuries

Injuries/trauma and emergencies is ranked fourth on the disease burden in Tanzania [62]. Victims of injuries/trauma often require blood transfusions to replace the massive amount of blood lost. Other recipients of blood transfusion include pregnant women, patients coming from surgery and those with anaemia. Pregnant women in African settings who need blood transfusions during or after delivery often suffer preventable deaths due to shortages of blood supplies [63]. Even though blood transfusion is considered a lifesaving intervention, it also exposes its recipients to blood-borne viral infections such as HIV/AIDS and Hepatitis B. In Tanzania the average HIV/AIDS prevalence among blood donors has been estimated to be 9 per cent [41]. Shortages of blood supply for transfusions and risks of disease transmissions make alternative options not requiring blood transfusions more attractive.

We found two pharmacoeconomic studies on Tranexamic acid (TXA) – an antifibrinolytic drug which reduces post-operative blood loss and transfusion requirements to injury victims [64]. TXA can reduce the risks of death due to bleeding by 21 percent if administered within three hours after injury [34]. For elective surgery, TXA reduces the requirement of blood transfusion by one-third and the volume per transfusion by one unit [33]. The incremental cost-effectiveness of administering TXA to bleeding trauma patients in Tanzania was 48 US$ per LY gained [20], while the incremental cost-effectiveness for surgical bleeding was US $ 93 per life saved [29]. Despite being reported to be very cost-effective in Tanzania, TXA injection is not on the national essential medicine list, but has recently been added to the WHO’s model list of essential medicines [65].

### Tuberculosis

TB is ranked sixth on the disease burden in Tanzania in spite of being recognized as having one of the most successful national TB programs in the world, with a treatment success rate of 88 per cent [37]. We found one relatively old economic evaluation study by Murray et al. which compared the cost-effectiveness of short-course versus long-course anti-TB chemotherapies. The study showed that short-course chemotherapy was less costly per death averted and per LY saved when compared to the long, 12-months chemotherapy for both hospital and ambulatory care [31]. The short-course strategy was found to be very cost-effective with incremental cost-effectiveness ratio of 1–4 US$ per life year saved. In areas with an organized healthcare system the short-course regimen increased the cure rate by a quarter when compared to the standard therapy [31]. Short-course chemotherapy was already introduced in Tanzania before the publication of the study conducted by Murray et al. However, our review of documents showed that the decision to adopt the use of short-course chemotherapy was grounded on evidence of better treatment outcomes at less costs shown by the short-course regimen in Tanzania [66].

### Cardiovascular diseases

Cardiovascular diseases are ranked tenth on disease burden and are the leading causes of mortality in elderly in Tanzania [40]. We found one pharmacoeconomic study by Robberstad et al. who explored the cost-effectiveness of 14 drug therapy combinations given to patients with cardiovascular diseases. They found incremental cost-effectiveness ratios ranging from 86 US$ to about 4,600 US$ per DALY saved, hydrochlorothiazide – a diuretic drug, being the most cost-effective option [22]. Review of the national essential medicine lists shows that many of the drug therapies they studied were already on the list but again without cost-effectiveness evidences for their selection.

### Rabies

About 5 people out of 100,000 die of rabies in Tanzania each year [67]. Deaths due to rabies, mostly from dog bites, can be prevented through post-exposure prophylaxis with anti-rabies vaccines. We found one pharmacoeconomic study by Shim et al., on anti-rabies vaccine for post-exposure prophylaxis which reported an incremental cost-effectiveness ratio of US$ 32 and US$ 27 per QALY gained, from societal and provider perspectives respectively [30]. This intervention is highly cost-effective and if scaled-up can avert 5,000 QALYs lost each year [30]. Anti-rabies vaccine has been on the national essential medicine list since 2007 [13], therefore the cost-effectiveness evidence provided by the study published by Shim et al. is too recent to have had influenced the decision to include the vaccine on the national essential medicine list.

### Use of pharmacoeconomic data from other settings

With only a few pharmacoeconomic analysis studies available for decision-makers in Tanzania, one is tempted to deploy economic evidences from studies conducted elsewhere. Cost-effectiveness studies are context specific and generalizations must always be done with great caution [68]. For example, healthcare costs depend on factors such as the structure and functioning of the healthcare systems, availability of healthcare resources and pricing mechanisms, which can vary from one setting to another. Effectiveness of drug therapies on the other hand depends on their utilization and performance in the real life conditions. Utilization of a drug depends on its acceptability and perceived side effects among the users. Therefore, before cost-effectiveness results from one setting can be applied to inform decision making in other settings, the relevance of such context specific factors should be evaluated by considering the impact of the differences on the results and conclusions. In well designed and well reported studies, such assessments can be accommodated with sensitivity and scenario analyses. We have seen that pharmacoeconomic studies conducted locally are scarce; therefore we argue that decision-makers in Tanzania sometimes can make use of pharmacoeconomic data available from similar African countries. However when the differences in context specific factors are large, or when the sensitivity of the results are insufficiently explored, such generalizations should not be made.

### Limitations of the study

The findings of this study are only based on information retrieved through systematic review of articles and relevant policy documents, and hence must be interpreted with care. We did not conduct any interviews to supplement the information we extracted from the policy documents which are neither readily nor consistently available in Tanzania due to logistic challenges. We therefore believe that our search may have not been exhaustive, and so there might be other policy documents containing relevant information related to this study which we did not manage to access.

## Conclusions

There are only a few pharmacoeconomic studies which have been conducted in Tanzania and which are useful to guide selection of essential medicines. The majority of these studies are narrow in scope hence do not correspond to drug selection challenges decision-makers are always confronted with in priority-setting decisions. We found little evidence suggesting that the existing pharmacoeconomic studies had impact on the selection and hence listing of drugs in the national essential medicine list. While we encourage more studies on pharmacoeconomic analysis to fill the existing gap, we also emphasise the importance to assess whether decision-makers in the drug selection committees have the ability to interpret and utilise cost-effectiveness evidence when assessing pharmaceuticals for inclusion in the treatment guidelines and essential medicine list. We also encourage Tanzanian health authorities to consider how health economic evidence should be applied more consistently in priority-setting decisions for selection of essential medicines.

## Competing interests

Both authors declare that they have no competing interests.

## Authors’ contributions

ATM and BR both conceived and designed the study. ATM carried out the reviews, data extraction and prepared the draft of the manuscript. BR supervised the review process and contributed on the manuscript writing. Both authors read and approved the final manuscript.

## Authors’ information

ATM holds a bachelor degree on pharmaceutical sciences and master’s degree on health policy analysis and management. ATM is an assistant lecturer at Muhimbili University of Health and Allied Sciences, Tanzania and currently he is pursuing a PhD program in Health Economics at the University of Bergen. BR is a professor in Health Economics at the University of Bergen.

## Pre-publication history

The pre-publication history for this paper can be accessed here:

http://www.biomedcentral.com/1472-6947/12/110/prepub
